# Text mining and manual curation of chemical-gene-disease networks for the Comparative Toxicogenomics Database (CTD)

**DOI:** 10.1186/1471-2105-10-326

**Published:** 2009-10-08

**Authors:** Thomas C Wiegers, Allan Peter Davis, K Bretonnel Cohen, Lynette Hirschman, Carolyn J Mattingly

**Affiliations:** 1 Department of Bioinformatics, The Mount Desert Island Biological Laboratory, Salisbury Cove, ME, USA; 2 Center for Computational Pharmacology, University of Colorado School of Medicine, Aurora, CO, USA; 3 Information Technology Center, The MITRE Corporation, 202 Burlington Road, Bedford, MA, USA

## Abstract

**Background:**

The Comparative Toxicogenomics Database (CTD) is a publicly available resource that promotes understanding about the etiology of environmental diseases. It provides manually curated chemical-gene/protein interactions and chemical- and gene-disease relationships from the peer-reviewed, published literature. The goals of the research reported here were to establish a baseline analysis of current CTD curation, develop a text-mining prototype from readily available open source components, and evaluate its potential value in augmenting curation efficiency and increasing data coverage.

**Results:**

Prototype text-mining applications were developed and evaluated using a CTD data set consisting of manually curated molecular interactions and relationships from 1,600 documents. Preliminary results indicated that the prototype found 80% of the gene, chemical, and disease terms appearing in curated interactions. These terms were used to re-rank documents for curation, resulting in increases in mean average precision (63% for the baseline vs. 73% for a rule-based re-ranking), and in the correlation coefficient of rank vs. number of curatable interactions per document (baseline 0.14 vs. 0.38 for the rule-based re-ranking).

**Conclusion:**

This text-mining project is unique in its integration of existing tools into a single workflow with direct application to CTD. We performed a baseline assessment of the inter-curator consistency and coverage in CTD, which allowed us to measure the potential of these integrated tools to improve prioritization of journal articles for manual curation. Our study presents a feasible and cost-effective approach for developing a text mining solution to enhance manual curation throughput and efficiency.

## Background

### The Comparative Toxicogenomics Database (CTD)

The etiology of many chronic diseases involves interactions between environmental factors and genes and proteins that modulate important physiological processes. Unfortunately, the mechanisms of actions of most chemicals and the etiologies of environmentally influenced diseases are not well understood [1]. We are developing CTD (http://ctd.mdibl.org) to promote understanding about the effects of environmental chemicals on human health [2,3]. To achieve this goal, we integrate manually curated data with select public data sets to provide a centralized, freely available resource for exploring cross-species chemical-gene and protein interactions and chemical- and gene-disease relationships.

**Figure 1:**
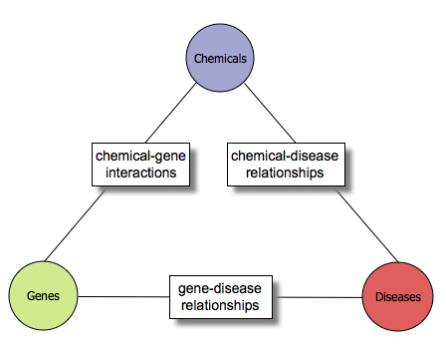
**CTD curated data relationships**. Biocurators capture three types of data relationships from the literature using controlled vocabularies, including chemical-gene interactions, and chemical-disease and gene/protein-disease relationships. These three relationships generate a chemical-gene/protein-disease triad that enables users to infer novel connections between all three actors.

CTD biocurators manually curate three types of data relationships from the peer-reviewed scientific literature: a) chemical-gene/protein interactions, b) chemical-disease relationships, and c) gene/protein-disease relationships (Figure 1). Currently, CTD provides over 178,000 interactions between 4,980 chemicals and 16,182 genes and proteins in 298 species as well as more than 5,600 chemical-disease and 8,900 gene/protein-disease relationships. By integrating curated data among chemicals, genes and diseases, novel transitive relationships can be inferred. CTD provides over 629,000 inferred gene-disease relationships and 172,000 inferred chemical-disease relationships that may be used to develop novel hypotheses about chemical-gene-disease networks. Additional molecular network and functional genomics insights can be determined through the integration of data sets from resources like the Gene Ontology (GO) [4] and Kyoto Encyclopedia of Genes and Genomes (KEGG) [5].

CTD is unique among other publicly available chemical, gene or disease databases, because: a) it focuses on environmental chemicals, b) it integrates manually curated and external data sets to specifically support understanding about the complex connections between chemicals, genes/proteins and diseases and c) it serves as more than just a data repository by supporting the generation of novel hypotheses about the environmental etiologies of human diseases through novel data integration and analysis tools [3,6,7]. The batch query tool allows users to retrieve associated data sets (e.g., GO annotations) for a list of chemicals, genes or diseases of interest. The VennViewer tool generates Venn diagrams for associated data sets for up to three chemicals, genes or diseases of interest (e.g., associated diseases for arsenic and bisphenol A). Additional tools in the pipeline will further enhance the capability of analyzing user-defined data sets in conjunction with CTD data sets.

### CTD curation

The CTD manual curation process is well defined and has been described previously in detail [2,3]. Here we provide a brief summary of the process. Journal articles are prioritized for curation by chemicals of interest. They are identified by querying titles and abstracts from MEDLINE using PubMed [8] and controlled chemical terms and synonyms from the National Library of Medicine's Medical Subject Headings (MeSH) [9]. Documents are ranked in date order (the default order from PubMed). Biocurators read abstracts and full-text articles from which they capture chemical-gene/protein interactions and disease relationships.

All curated interactions and relationships are captured using controlled vocabularies and ontologies to maximize consistency among biocurators, ensure reproducible data retrieval by users, and enable integration of CTD data with other databases. The CTD ***chemical ***vocabulary derives from a modified subset of the chemicals and supplementary concepts in the "Drugs and Chemicals" category of MeSH. For ***genes and proteins***, CTD uses official gene symbols and names from the National Center for Biotechnology Information's (NCBI) Entrez-Gene database [10]. Where possible Entrez Gene entries representing orthologs are merged into a single, cross-species gene entity in CTD (e.g., CTD's AHR gene comprises the *Mus musculus *Ahr and *Homo sapiens *AHR, among others). Curators use these cross-species genes in CTD to capture chemical interactions and disease relationships. The CTD ***disease vocabulary ***uses terms from MeSH and OMIM [10]. CTD ***interaction types ***are described using terms from a hierarchical vocabulary of 50 diverse relational terms (*e.g*., "binding," "phosphorylation") developed by CTD curators. ***Organisms ***in which chemical-gene interactions are curated are specified using terms from the Eumetazoa portion (vertebrates and invertebrates) of the NCBI Taxonomy database [10].

**Figure 2:**
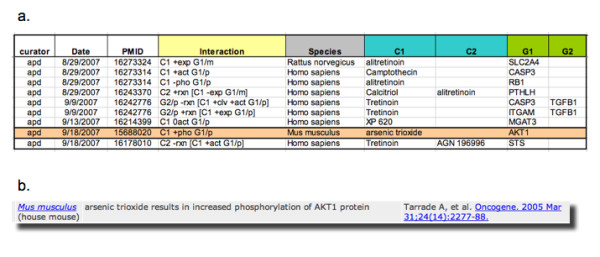
**Documentation of curated data**. a) Currently curated data are captured using controlled vocabularies in Excel spreadsheets that include: Curator ID, date of curation, PubMed identification number, interaction (designated using a CTD coding schema), species in which the interaction was observed, interacting chemical, interacting gene/protein, associated diseases (not shown) and author contact information for follow-up purposes (not shown). b) Codes used to capture interactions are translated into readable sentences for the public web application.

Curators are trained by a lead curator, using a manual that provides detailed instructions for identifying and capturing chemical-gene interactions and disease relationships. Currently data are captured in Excel spreadsheets that include the following data: Curator ID, date of curation, PubMed identification number, interaction, species in which the interaction was observed, interacting chemical, interacting gene/protein, associated diseases and author contact information for follow-up purposes (Figure 2a). Curated data are then loaded into a database for quality control review prior to public release. Interactions are captured in the spreadsheets using a CTD-defined shorthand or code that is translated into full sentences in the public web application (Figure 2b).

### Curation challenges

High quality manual curation of the scientific literature is a common bottleneck to populating biological databases. It is challenging to keep pace with the increasing volume and scope of published biological data, although CTD curation efforts have become increasingly efficient. To further enhance the efficiency of curation and evaluate our coverage of the published literature for priority research areas we embarked on the project described here. The goals of this project are to: a) generate a baseline analysis of CTD manual curation; b) develop a prototype text-mining application that would address CTD curation needs by identifying chemical, gene/protein, and disease terms in journal articles to rank them effectively for manual curation, and to provide interactive tools for extraction of chemical-gene-disease interactions; and c) assess the future impact of this workflow on curation and data coverage in CTD. CTD staff developed prototype applications for the document-ranking task in collaboration with members of the Information Technology Center of the MITRE Corporation and the University of Colorado School of Medicine's Center for Computational Pharmacology. Based on results presented in this report, future studies will focus on implementing our text-mining pipeline into the CTD manual curation workflow.

## Results

To assess the extent to which text mining could improve the coverage and efficiency of curation, we performed a baseline study of the current manual curation workflow. This assessment allowed us to identify where in the workflow the prototype could be applied. Two prototypes were developed and evaluated using a gold standard of manually curated interactions and relationships from CTD.

### Baseline analysis of CTD manual curation

To determine areas of the CTD curation process that could benefit from text mining, we established baselines for curation rates and consistency. Three CTD biocurators were given an identical set of 112 journal articles identified using PubMed along with terms for three different chemicals. Biocurators were instructed to curate and record the time they spent on each article. Results are reported in Table 1.

**Table 1:** CTD manual curation metrics

**Data**	**Biocurator 1**	**Biocurator 2**	**Biocurator 3**	**Average**
**Total no. articles examined**	112	112	112	112
**No. articles curated (%)**	57 (51)	74 (66)	69 (62)	67 (60)
**No. articles rejected (%)**	55 (49)	38 (34)	43 (38)	45 (40)
**Time spent reviewing articles**^***a***^	1331	893	2263	1496
**Time spent on curatable articles (%)**	1198 (90)	822 (92)	2133 (94)	1384 (93)
**Time spent on rejected articles (%)**	133 (10)	71 (8)	130 (6)	111 (7)
**Curation rate (+/- SD) **^***b***^	21.0 (31.1)	11.1 (13.1)	30.9 (52.9)	20.7
**Rejection rate (+/- SD) **^***c***^	2.4 (3.4)	1.9 (3.1)	3.0 (4.4)	2.5
**Total data extracted**^***d***^	828	2330	3039	2066
**Data per curated article (+/- SD)**	14.5 (34.4)	31.5 (143.7)	44.0 (209.8)	30.8
**Data extraction rate (+/- SD)**	0.5 (0.3)	1.4 (1.7)	0.6 (0.6)	0.8

^***a***^All times and rates were recorded or calculated in minutes

^***b***^Curation rate = Time spent per curated article. SD = standard deviation.

^***c***^Rejection rate = Time spent per rejected article.

Total data extracted = total number of chemical-gene, chemical-disease, and gene-disease interactions.

^***d***^Data extraction rate = macro-average of individual rates of the number of interactions for each curatable article.

On average, biocurators rejected 40% of the 112 journal articles as not having curatable data. Although the rejected articles contained both chemical and gene terms, they were typically rejected because they did not describe an actual chemical-gene interaction. Rejected articles were easily identified and on average, biocurators only invested 7% of their time on them (average of 2.5 minutes per rejected article). Biocurators averaged 21 minutes per curatable journal article, including those for which the full text was consulted. As indicated by the differences in the average time spent per curatable article, curation rates varied by individual biocurator. The large standard deviations were due to the fact that some journal articles had only a few interactions and took only a few minutes to curate, while others had many interactions and took much longer; four articles in this set had between 100 and 1,000 curated interactions. The longest average time spent on an individual article from this set was 166 minutes, but the time investment resulted in curation of a substantial amount of data. An average of 31 interactions were extracted from each curated article, at an average rate of 0.8 interactions per minute. These data establish a critical baseline for future studies in which the impact of integrating text-mining tools on the CTD manual curation process will be measured.

#### Inter-Biocurator Agreement

To determine whether text mining could enhance precision and recall of data curation, we measured a baseline for inter-biocurator consistency by calculating how often biocurators captured the same chemical-gene interactions from the same journal article using the set of 112 articles described above.

We performed the analysis in two steps. In Step 1, we assessed curator consistency about whether or not to curate a particular article. Table 2 demonstrates that the curators agreed on the disposition of 86/112 articles (77%) and had an average pair-wise agreement of 85%. In Step 2, we compared agreement between each curator and a "gold standard" set of interactions, averaged over all the documents curated by that curator. To prepare the "gold standard," the lead biocurator validated any interaction where curators disagreed, labeling each interaction as correct or incorrect for each curated paper. This set of correctly labeled interactions enabled us to compare each curator's results to the gold standard and to calculate precision and recall on a per-document basis; precision and recall were calculated only for those documents curated by that curator. The results (Table 3) showed that 91% of the interactions extracted by CTD biocurators were judged by the lead curator to be correct (average precision = 0.91). Average recall was 0.71.

**Table 2:** Degree of consensus to curate

	**Biocurators**^***a***^ **1+2+3**	**Biocurators**^***a***^ **1+2**	**Biocurators**^***a***^ **2+3**	**Biocurators**^***a***^ **1+3**	**Pair-wise average**
**No. articles agreed to curate**	52	57	65	52	58
**No. articles agreed to reject**	34	38	34	38	37
**No. articles with agreement**	86	95	99	90	95
**No. articles with disagreement**	26	17	13	22	17
**No. articles examined**	112	112	112	112	112
**Consensus (%)**	0.77	0.85	0.88	0.80	0.85

^***a***^Numbers represent each of the three CTD biocurators.

**Table 3:** Precision, recall, and f-measure of CTD manual curation

**Data**^***a***^	**Biocurator 1**	**Biocurator 2**	**Biocurator 3**	**Average**^***e***^
**No. articles examined**	53	68	65	62
**Precision**^***b***^	0.90	0.97	0.86	0.91
**Recall**^***c***^	0.62	0.79	0.71	0.71
**F1-measure**^***d***^	0.70	0.85	0.75	0.77

^***a***^Only curated chemical-gene interactions were analyzed (disease interactions were not considered); consequently, these numbers differ slightly from those reported in Table 2.

^***b***^Precision = Correct interactions for individual biocurator/Total interactions for individual biocurator.

^***c***^Recall = Correct interactions for individual biocurator/Total correct interactions from all biocurators.

^***d***^F1-measure is the harmonic mean of precision and recall = (2 × Precision × Recall)/(Precision + Recall). Precision, Recall, and F1-measure were macro-averaged over the set of articles curated by that curator.

^***e***^Calculated using data from three biocurators.

### Assessing performance of prototype text-mining applications

Assessment of the potential value of our prototype text-mining tools focused on: a) effectiveness of identification of "actors" in abstracts of biomedical journal articles where an actor was defined as a chemical, gene/protein or disease of relevance to the CTD project; and b) effectiveness of document ranking to help prioritize journal articles for manual curation.

#### Actor Identification

Tools for identifying chemical (OSCAR 3 and MetaMap; [11-13]), gene/protein (ABNER and MetaMap; [14]) and disease (MetaMap) terms were identified and integrated into a prototype workflow. Experiments described here were compared against a set of manually curated data from 1,600 journal articles in CTD for 10 chemicals. The effectiveness of the text-mining tools was evaluated to determine the proportion of manually curated actors that were successfully identified (i.e., chemicals, genes, diseases participating in CTD curated interactions for those documents). Overall the tools identified 80% of curated actors (74% for curated gene actors, 94% for curated chemical actors, and 51% for curated disease actors) from the 1600 manually curated set of articles ('gross actor identification ratio'). Because the text-mining tools were limited to searching only the titles and abstracts of journal articles, whereas much of the manually curated data derived from the full text, this calculation understated the effectiveness of the tools. Consequently, a second ratio, the adjusted actor identification ratio, was developed to account for this added complexity. Using the adjusted ratio 92% of the actors that were manually curated from the titles and abstracts of journal articles were identified by the text-mining tools (93% for curated gene actors, 99% for curated chemical actors, and 80% for curated disease actors). The average response times of the ABNER and OSCAR3 text-mining tools were each approximately one second per abstract; the MetaMap tool was 1 minute, 17 seconds.

#### Document Ranking

**Figure 3:**
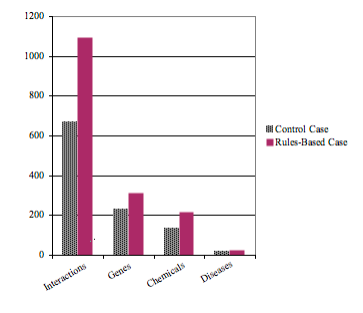
**Rules-based ranking of articles enhances yield of curated data**. When ranked using the rules-based application vs. PubMed ordering (control case), the top 10% of articles would result in an increased yield of curated data; specifically 426 more chemical-gene interactions, comprising 82 additional genes, 81 additional chemicals and 5 more diseases.

A goal of a text-mining tool for CTD was to identify relevant journal articles and prioritize them for manual curation. There were two important factors for gauging the effectiveness of ranking journal articles: a) the extent to which the system identified and ranks *relevant *documents (i.e., those containing one or more curatable interaction), more highly than non-relevant documents; and b) the extent to which the system ranked documents that were information-rich more highly than those that were not (e.g., a document containing 20 curatable interactions should be ranked more highly than a document containing only a single curatable interaction). These two issues are critical because many times there are more documents available for a given area of interest than can be realistically manually curated. Currently, we impose cut-off criteria available through the PubMed query interface (e.g., publication date). Instead we would like to use a more informed ranking method to ensure that we are achieving more complete coverage of curated data in CTD while also optimizing biocurator productivity. Mean average precision (*MAP*) was used to quantify the ability of a ranking system to rank relevant documents more highly than non-relevant documents and a correlation coefficient (Pearson's product moment correlation coefficient, or *R*) was used to correlate the ranking of articles with data richness. The text-mining study found that the *MAP *was 63% for the baseline case (the document ordering from the original query to PubMed); this rate is actually quite high and reflects the skill of the CTD biocurators in effectively employing the PubMed search capabilities. Nonetheless, the text-mining tools improved significantly upon the default ordering: *MAP *increased to 72% for the Lucene-based application, and 73% for the rule-based application - an improvement of almost 16% over the baseline case. The correlation between PubMed ordering of documents and the richness of curated interactions was 0.14. Correlations more than doubled with the text-mining tools: 0.32 for the Lucene ranking method and 0.38 for the rule-based ranking method. Figure 3 illustrates the important implications of improved ranking on the resulting curated data. When ranked using the rules-based application vs. PubMed ordering (control case), curation of the top 10% of the 1,600 articles would result in 426 more chemical-gene interactions, including 82 additional genes, 81 additional chemicals and 5 more diseases.

The caveat to these results is the tools "overtagged" the articles: only 36% of the tagged actors participated in curated interactions (38% of genes, 37% of chemicals and 11% of disease terms); altogether, 64% of the tagged terms had no curatable interactions associated with them; we describe these as 'false positive actor mentions'. There are a number of reasons for these false positives, including legitimate mentions of genes/disease/chemicals that are not involved in a curatable interaction in the paper; gene names that are synonyms of chemicals (e.g., ROS, PGE2); and many short names or symbols (e.g., AS) that are synonyms of genes (e.g., HLA-B) and chemicals (e.g., ammonium trichloro(dioxoethylene-O, O'-)tellurate) and are confusable with commonly used adverbs, prepositions or conjunctions in English. In order to create a tool that curators can productively use to extract interactions from articles, it will be important to reduce these false positive actor mentions.

#### Indomethacin case study

**Figure 4:**
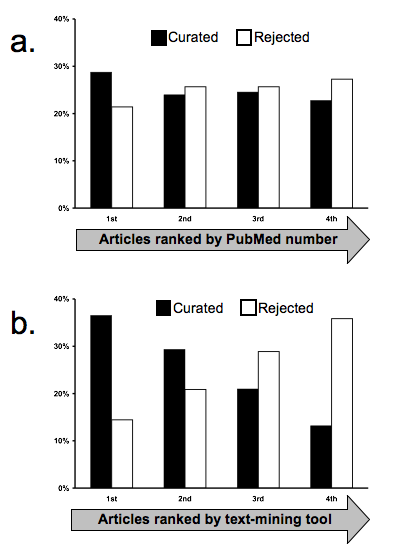
**Text mining improves the ranking of journal articles for curation**. A test set of 354 articles slated for curation were first ranked by two different methods: (a) via each article's PubMed identification number in descending order (which typically reflects the publication date from newest to oldest paper) and (b) via the rank order determined by our rule-based text-mining application. The articles were then reviewed by a biocurator who determined that 167 of the papers contained relevant data (curated, black bars) while 187 of them did not (rejected, white bars). For presentation, the 354 articles are grouped into progressive quartiles (1st, 2nd, 3rd, and 4th) each containing 89 papers. The overall percent of total curated papers (167) vs. rejected papers (187) are shown distributed over each quartile. The text-mining tool (b) effectively ranked the more relevant papers into the first and second quartile and the less relevant papers to the third and fourth quartile compared to the less informed criteria of PubMed identification numbers (a).

Based on the promising results for actor identification and document ranking described above, we evaluated the potential for our rule-based text-mining tool to identify and rank a another test set of articles for curation. To do this, we retrieved data from MEDLINE for indomethacin, a chemical for which we had previously curated 73 journal articles. This time we used a broader PubMed query to evaluate the performance of our text mining tools in comparison with our existing curated data for this chemical. We identified 1,138 journal articles of which our 73 curated articles were a subset. Based on identification of actors by our text-mining actor recognition tools, we filtered the 1,138 results to a set of 354 journal articles that contained gene/protein actors *not currently associated with indomethacin *in CTD. These 354 journal articles were then ranked and reviewed by our lead biocurator who determined that 167 (47%) articles contained curatable data for indomethacin. Notably, there was a strong correlation between the ranking of these articles by the text-mining tools and whether the articles contained curatable data (Figure 4). This correlation was not seen with our previous method for ordering articles for curation, namely the descending order of PubMed identifiers, which typically reflect publication date. Overall, MAP improved from 54% under the baseline case to 68% under the rule-based case. Subsequent curation of these 167 articles indicated that text mining effectively identified articles with novel data for indomethacin including 72 genes, 192 chemical-gene interactions, and 60 diseases. These results have important implications for future implementation in our curation process regarding the potential of text mining to help assess the existing scope of curated data in CTD, identify novel data for curation and inclusion in CTD, and effectively prioritize documents for curation.

## Discussion and Conclusion

A major goal of CTD is supporting development of novel hypotheses about the complex relationships between chemicals, genes/proteins and diseases. In most cases, such complex relationships have not yet been elucidated. To support hypothesis development we have taken a reductionist approach by curating individual relationships and integrating them in ways that have the potential to reveal previously unidentified, complex connections. As with other manually curated resources, these efforts are challenged by the increasing scope and volume of data being published. In this study we explored whether we could find a solution that would merge manual curation, which is highly accurate but time consuming, with text mining, which is scalable but error-prone [15].

There are still relatively few examples of working tools inserted into a biocuration pipeline, despite a number of assessments of text mining applied to biomedical curation [16]. The Textpresso software [17-19] has been applied successfully to a number of model organism databases, as well as to other kinds of curated databases. Textpresso uses multiple ontologies to create lists of terms that can be identified in running text. Users can construct their own search using these ontologies. Other tools in active use are ProMiner [20] and RLIMS-P [21], which extract from articles gene/protein terms and phosphorylation events, respectively. The European Bioinformatics Institute's Whatizit software [22] provides, among other things, a retrieval/search engine for PubMed abstracts, identifying molecular biology terms in a number of categories and linking them to publicly available databases. There are other similar special purpose search tools, such as iHOP [23] or Chilibot [24] that focus on identifying specific types of biological entities (genes, proteins) and their relations. However, none of these solutions addressed the specific needs of the CTD curation workflow.

We report here a novel approach to building a text-mining solution for our publicly available database that began with establishing baseline metrics for our current curation process. From the results of this analysis we identified areas in which text mining could add value to the CTD data curation workflow. We determined that identification of journal articles from MEDLINE for potential curation yields a large percentage (40%) of articles that are not curatable, but that rejection of these journal articles consumed a relatively small percentage of biocurator time (7%). These results suggest that biocurator identification of relevant journal articles is efficient; however, more effective identification and ranking of relevant journal articles by a text-mining application would further maximize productivity and increase the quantity of data curated in CTD. We performed inter-biocurator agreement studies to determine how well biocurators agreed when curating an article, to provide an upper bound for performance of text mining. We calculated that precision for curated actors was high (average, 0.91) and recall was lower (average, 0.71). This is consistent with results reported by Camon et al. [25], where an inter-curator agreement study for GO annotation of proteins showed high precision (few incorrect GO annotations, 94%), but variation in the depth or exhaustiveness of curation, leading to missing annotations and lower recall (72%).

Based on our curation needs assessment, we developed two prototype text-mining applications. One application was built using the Lucene search engine API [26] whereas the other was rule-based. Both applications integrated a set of publicly available actor identification tools for chemicals, genes/proteins and diseases. We leveraged the large corpus of curated data from CTD to construct a gold standard data set that included over 6,000 curated actors from 1,600 journal articles describing 10 different chemicals. This gold standard data set was used to examine the potential value of these text-mining applications to enhance CTD curation.

The two performance areas of particular interest to us were: a) how effectively the actor recognition tools identified terms of interest (chemicals, genes/proteins, diseases) in journal articles; and b) how reliably the applications could rank documents such that ranking could assist with prioritizing articles for manual curation. Actor identification was very effective (80% of all curated actors identified), particularly when adjusted for actors that seemed to be found only in the abstracts of journal articles from our control data set (92%). Interestingly, text mining and manual curation share many of the same challenges, not the least of which is gene name identification and inconsistency in nomenclature use [15]. Given these challenges, our actor identification results were particularly gratifying.

The new document ranking strategies showed great promise for effectively identifying information rich, relevant journal articles. Based on CTD curation experience, we identified a range of criteria against which to rank documents. Both ranking strategies effectively improved the ranking over the baseline case, although our rule-based application slightly outperformed Lucene in every metric. In addition, the rule-based application enabled us to exercise greater control over document scoring and ranking because it was entirely customized for CTD curation unlike Lucene, which is customizable to an extent, but was designed to be general-purpose. Similar results were found in a new test case for indomethacin journal articles, where we demonstrated that the text-mining applications ranked articles with curatable data more highly that those without curatable data. This correlation is a significant finding because it shows that these tools will allow us to cast a wider net when identifying potential journal articles for curation because we will be able to prioritize articles for curation more knowledgeably and maximize the productivity of biocurators by presenting them with a subset of information-rich journal articles that are more likely to contain curatable data (Figure 3). Specifically, our rules-based application will enable us to more effectively identify information-rich articles and thereby capture more data from an equivalent number of articles. Effective actor identification will also enable better assessment of our data coverage for particular chemicals, genes, or diseases and consequently identify data that may be missing or in need of updating in CTD using an approach similar to our indomethacin study. Finally, although we might maximize information retrieval by mining the full text of journal articles rather than just abstracts, our indomethacin results corroborate a recent report indicating that reviewing abstracts as an indexing unit may be comparable to reviewing the full text of articles [27]. Therefore, at least for ranking, attempting to overcome the many additional challenges associated with mining full-text articles may not yield substantially better results than mining abstracts.

We are greatly encouraged by the results of our prototype text-mining applications. Our results demonstrate that it is possible to integrate "off-the-shelf" tools to provide significant value to a biocuration workflow. We applied a method based on inter-biocurator agreement of manual annotation to identify where to insert text-mining tools into the curation workflow to maximize pay-off. We were able to assemble quite rapidly a text-mining pipeline by using freely available entity recognition software; we developed two ranking strategies and evaluated their performance. We estimate that the actual tool acquisition and integration took only a few staff weeks; the bulk of the time on the project was spent in creation of a baseline for comparison, and in evaluation of the performance of the two ranking approaches (Lucene and the rule-based approach). While we chose entity recognition systems based on the specific CTD application, we believe that this approach can be extended both to handle other parts of the CTD curation pipeline, and to other curation applications. To further improve our results, we will evaluate the impact of optimizing weighting criteria using multivariate analysis; modifying requirements for retrieving actor terms (e.g., length of words, removal of common confusable English words); and using additional criteria including inclusion of CTD action terms in the search for actors, and extending analysis to the full text of journal articles. We will also look carefully at certain aspects of the EBI's Whatizit software. Although Whatizit employs all of the major recognition tools that we selected for integration into our prototypes, we will specifically evaluate their disease tools in an attempt to mitigate the extended response times we experienced during MetaMap processing.

**Figure 5:**
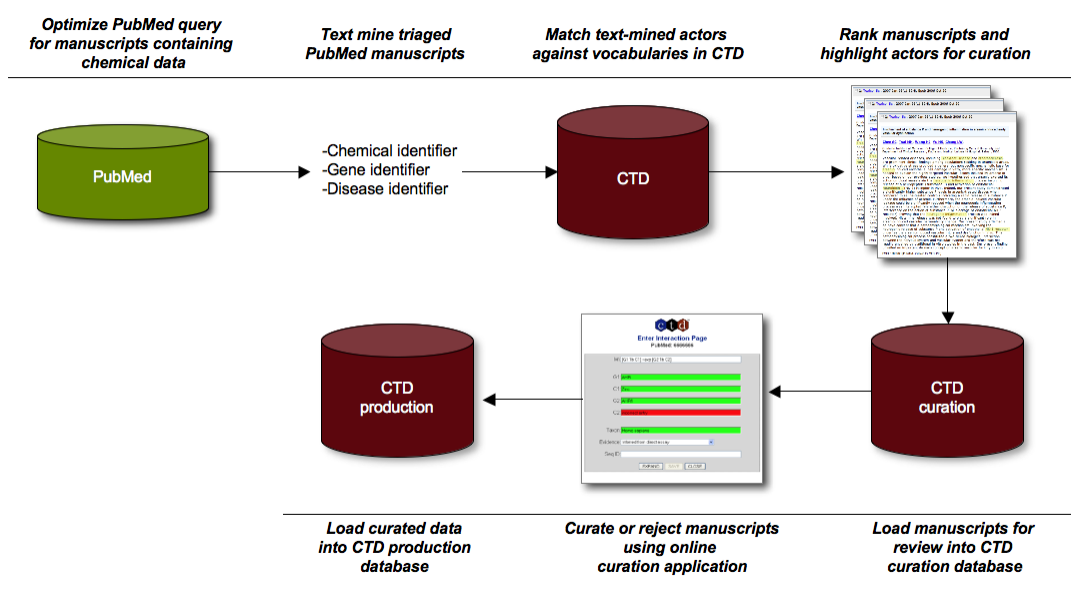
**Future CTD manual curation workflow**. Articles will continue to be identified for curation using PubMed and chemical terms of interest. Articles will be text mined using chemical (OSCAR 3), gene (ABNER) and disease (MetaMap) identifiers as described. Actors identified by text mining will be matched against vocabularies in CTD and journal articles without matches will be removed. Remaining journal articles will be ranked and loaded into the CTD curation database. Biocurators will curate or reject journal articles using an online application tool that is integrated with the CTD curation and production databases. Curated data will be approved and loaded into the CTD production database.

Based on our results we are planning modifications to our existing manual curation process that are illustrated in Figure 5. MEDLINE will continue to be searched using PubMed for priority chemical terms and their synonyms. Resulting journal articles will be text-mined using actor identifiers for chemicals, genes and diseases, as well as action terms. These actors will be cross-referenced with corresponding vocabularies in CTD. Corroborated actors will be highlighted, and journal articles will be ranked and loaded into the curation database. Biocurators will review and curate highlighted journal articles using an online curation application with real-time quality control measures. Curated data will be loaded into our production database on a real-time basis, and made available to the public monthly. New baseline assessments will be made to accurately determine the impact of incorporating text mining with the CTD curation process.

This report provides a compelling demonstration that text mining for the biological literature has matured to the point where it is feasible and cost-effective to insert text-mining tools into the curation pipeline to improve both curation throughput and quality. Our approach, consisting of baseline creation, tool integration and evaluation can be readily generalized to other applications both within CTD and for other curated databases.

## Methods

### CTD manual curation analysis

CTD data is curated by three biocurators, all of whom have a Ph.D. in the biological sciences, have been with CTD for more than one year, have undergone thorough training in database policies, and had prior experience as biocurators for other databases, making them highly competent and experienced professionals. To assess a baseline analysis of CTD curation, the three biocurators were presented with the same list of 112 journal articles identified from MEDLINE using PubMed, and instructed to curate them at their normal pace according to CTD curation policies and record the time spent on each article. They read the title and abstract of each article and had the option to read and curate the full text. The full text was generally consulted to resolve ambiguities in the abstracts (e.g., official gene symbol, species). Assigning the same journal articles to all three biocurators provided replicates for data analysis to identify fluctuations in curation rates and curation variability sometimes observed between data sets for different chemicals. Journal articles were identified using our standard protocol, which consisted of iterative PubMed queries that included: a) a target chemical, b) terms such as "gene" OR "mRNA" to enrich for journal articles that co-mention a chemical and gene [2], and c) recent publication date restrictions to retrieve data sets that are manageable to query and more frequently use official gene nomenclature. The baseline assessment occurred during December 2008. Two of the biocurators were unaware that they were curating the same set of articles. Curated chemical-gene interactions from the 112 articles were evaluated by the lead biocurator for correctness (correct chemical-gene interaction from the correct species). Data precision was calculated in the usual way as the number of correct interactions contributed by a curator divided by the number of interactions returned by that curator. To calculate data recall, we combined correct interactions returned by any of the curators to create a set of possible correct interactions for each document; these were then validated by the lead curator to create the gold standard. Recall was calculated in the usual way as the number of correct interactions returned by a curator for a given document, divided by the number of possible correct interactions for that document. These per-document precision and recall figures were then averaged across the set of documents curated by each curator, to derive a (macro-averaged) precision and recall per curator, as well as f-measure.

### Text-mining applications and analysis

#### Text-mining applications

We assembled two document ranking applications. Both were built in a modular fashion, utilizing publicly available tools for components of the workflow. One application was built using the Lucene search engine API [26]. The other application was a rule-based system constructed to search input documents for certain features and increment a weighted score for every feature found. Both applications made use of three publicly available named entity recognition systems, which are computer applications that locate mentions of some semantic class or classes of data types in texts. For example, given an input of "analysis of glutaredoxin mutant strains revealed that only those lacking the grxA gene are impaired in arsenic resistance" [28] a gene/protein named entity recognition system would locate the strings *glutaredoxin *and *grxA*, whereas a chemical named entity recognition system would locate the string *arsenic*.

Since CTD targets genes/proteins, chemicals, and diseases for curation, we selected three named entity recognition systems that were optimized for these specific semantic classes.

• *OSCAR 3 *was used to identify chemicals [11,12]. OSCAR 3 was selected because of the very small number of available chemical named entity recognition systems, it covers the broadest range of chemicals, and also because when possible, it returns a normalized identifier for chemicals that it locates in text.

• *ABNER *was primarily used to identify genes/proteins [14]. ABNER is a popular gene/protein named entity recognition system both because it achieves reasonable performance and because it is well-engineered, making it easy to incorporate into complex text-mining applications. Other alternatives exist, such as LingPipe and BANNER [29].

• *MetaMap *was used to identify diseases [13]. MetaMap was selected because it is the industry standard for locating mentions of clinical concepts, including diseases. We also note that MetaMap has been successfully used in genomics-domain applications in a number of instances [30]; in our case, it was also a useful supplement to both ABNER for locating gene/protein mentions, and to OSCAR 3 for chemical recognition.

Each of the entity recognition systems were integrated into the individual applications as-is. The entity recognition systems were used in the following order: genes, chemicals, and diseases. For actor identification reporting purposes, each tool was evaluated independently, i.e., if the same entity were recognized by both the gene and chemical entity recognition systems, and the entity was actually a curated chemical, correct recognition would be counted for chemical recognition system actor identification reporting.

One document ranking application was built using the Lucene search engine API [26]. Lucene was selected because its clean interface, well-engineered back end, and good performance have made it one of the most common tools used for building information retrieval engines (e.g., Wikipedia). It functions well enough that it is typically used out-of-the-box to establish a performance baseline for biomedical information retrieval systems.

The other application was a rule-based system constructed to search input documents for certain features (e.g., gene, chemical, and disease names), and increment a weighted score for every feature found. In contrast to Lucene, which ranks documents as more or less likely to be relevant to the given information need using a formula based on the relative rarity of these features, the rule-based system ranks them by a more finely controllable scoring formula. Rule-based systems can perform very well for document classification problems, and rating a document as relevant to an information need or not can be considered as a kind of document classification task.

#### Expert curated data set

Approximately 1,600 journal articles previously manually curated for CTD were used as a baseline data set, or "gold standard," to evaluate the performance of our prototype text-mining applications. These documents were a subset of the approximately 25,000 documents reviewed by biocurators since CTD manual curation began in 2005. The 1,600 documents contained 6,664 curated actors, including chemicals, genes, and diseases and represented data for 10 different priority chemicals: urethane, aspartame, 2-acetylaminofluorene, cyclophosphamide, indomethacin, aniline, raloxifene, amsacrine, phenacetin, and doxorubicin.

#### Evaluation of actor identification

Our information retrieval applications were used to identify actors in the titles and abstracts of journal articles within our control data set. Tagged terms identified by the applications were assessed for relevancy by comparing against CTD controlled vocabularies (including synonyms) for chemicals, genes and diseases. This had the effect of collapsing all synonymous mentions of an actor within a single document to the CTD defined term for that actor. Tagged terms not found within the CTD vocabularies were eliminated from subsequent analysis and ranking. Effectiveness of actor identification was evaluated by calculating the gross and adjusted actor identification ratios. We calculated the gross actor identification ratio as the number of curated actors (or their synonyms) identified by the text-mining tools divided by the number of curated actors in CTD. We also calculated 'adjusted actor identification ratio' to take into account the fact that the text-mining tools only searched titles and abstracts. This was calculated as the number of curated actors (or their synonyms) identified by the text-mining tools divided by the number of curated actors whose terms or synonyms were found in the abstract. However, this measure is imperfect in that the adjustment will overlook a phrase in an abstract such as "...causes cancers of the breast..." which could be curated to the term "breast neoplasms" by a biocurator, but would not be identified by the tools because "cancers of the breast" is not a synonym for "breast neoplasms". In this case, the adjusted actor denominator will incorrectly assume that the mention is not in the abstract, leading to a potential overestimate of performance. Both gross and adjusted ratios are calculated using macro-averaging - averaging the ratios for each article rather than the aggregate raw actor counts. This method is used to moderate the impact of large microarray-based journal articles for which abundant data is often only available in the full text.

#### Document ranking

A goal of text mining development for CTD curation was ranking documents in a manner that would help prioritize journal articles for manual curation. The curation group established ranking criteria. Each criterion was weighted in a way that seemed logical based on collective curation experience and optimized the mean average precision (MAP) score of the articles. The same basic ranking criteria were used for both the Lucene-based and the rule-based applications. Generally, documents that met the following criteria were weighted more highly than documents that did not:

• Target chemical mentioned in abstract title;

• Frequency with which the target chemical was mentioned in abstract;

• Target chemical was mentioned in first 2 sentences;

• Target chemical was mentioned in the last 2 sentences;

• Target chemical was included in PubMed MeSH annotation;

• Abstract was published in one of the following high priority journals: Nature, Science, Environmental Health Perspective, Toxicological Sciences, Cell, Journal of Biological Chemistry;

• Abstract referred to microarray data (e.g., "microarray" was mentioned in the abstract);

• Frequent mention in the abstract of specific genes in the CTD controlled vocabulary;

• Co-occurrence of action terms and actors in the same sentence.

The formulas used for document ranking are provided as Additional file 1.

*MAP *and correlation coefficients were used to evaluate the effectiveness of ranking and weighting of criterion. *MAP *measures the ability of text-mining tools to effectively identify and rank relevant documents by ranking those documents that appear to have relevant interaction data more highly than those that do not. It is calculated as the average of the precision at each point in the list of returned documents where the document at that point was relevant. The overall average precision was calculated as the average across the sets of documents for all 10 test chemicals [31]. A document was considered relevant if it contained curatable data. Correlation coefficients (Pearson's product moment correlation coefficient, or *R*) that measured the correlation of text-mining document ranking to the document ranking based on interaction richness (i.e., the number of interactions that were curated) were calculated for each ranking method and were defined by the number of curated interactions associated with articles.

#### Refining Actor Identification

A planned product of a text-mining system is an online curation application that will present abstracts to biocurators in which relevant actors are highlighted and hyperlinked back to CTD. In order to hyperlink a text-mined term, the text-mining tool would have to identify it; the term must exist verbatim in the abstract; and the term, or a synonym of the term, must exist among the respective CTD's controlled vocabulary. We assessed a 'false positive ratio' for our control data set and defined this ratio as the number of term mentions identified by the text-mining tools that were not actually involved in a curated interaction, divided by the total number of hyperlinked actors.

## List of Abbreviations Used

The following abbreviations were used herein: API: refers to application programming interface; CTD: refers to the Comparative Toxicogenomics Database; GO: refers to the Gene Ontology; GOA: refers to the Gene Ontology Annotation database; KEGG: refers to the Kyoto Encyclopedia of Genes and Genomes; MAP: refers to mean average precision; MeSH: refers to Medical Subject Headings; NCBI: refers to the National Center for Biotechnology Information; OMIM: refers to the Online Mendelian Inheritance of Man.

## Competing interests

The authors declare that they have no competing interests.

## Authors' contributions

TCW evaluated, modified and implemented text-mining workflow and conducted analyses of CTD datasets. APD collected control study data sets, conducted manual curation analyses and evaluated text-mining experimental results. KBC and LH provided consultation on identification of appropriate software tools, implementation strategies, and metrics. CJM initiated the collaborative study and participated in its design and coordination. All authors contributed to the writing and review of the final manuscript.

## Supplementary Material

Additional file 1 **Document Ranking Algorithms**. Details are provided of the rule-based and Lucene ranking algorithms. Click here for file
