# Supplementary material for: Text mining and manual curation of chemical-gene-disease networks for the Comparative Toxicogenomics Database (CTD)
Source: BMC Bioinformatics. 2009 Oct 8;10:326. doi: 10.1186/1471-2105-10-326 (PMC2768719; doi:10.1186/1471-2105-10-326)
Supplement: Additional file 1 — Document Ranking Algorithms. Details are provided of the rule-based and Lucene ranking algorithms. [file 1471-2105-10-326-S1.PDF]

Additional Materials

A variety of criteria and accompanying weights were used to rank documents with the rule-based and Lucene-based ranking algorithms described in this report. Those that yielded the best results when compared against our manually curated data were used for this study and are summarized below. These criteria were based on the curators' experience and intuitions, but there was no formal method used for recalibrating or deriving these weights

Rule-Based Ranking Algorithm

The ranking score for the rules-based application is based on an aggregation of the following factors:

- 1 point for abstracts appearing in one of the following priority journals:
  - Nature
  - Science
  - Environment Health Perspectives
  - Toxicological Sciences
  - Cell
  - The Journal of Biological Chemistry
- 2 points for each gene, chemical, and disease identified by the recognition tools and also resident in CTD as term or synonym, if the abstract contains both genes and chemicals; otherwise, 1 point is provided for each gene, chemical, and disease identified by the recognition tools and also resident in CTD as term or synonym.
- 4 points for each action term stem appearing in the abstract, if the abstract contains both genes and chemicals; otherwise, 1 point for each action term stem. Action terms include: *binding, activity, localization, expression, mutagenesis, stability, splicing, folding, transport, uptake, secretion, export, degradation, cleavage, glycosylation, N-linked glycosylation, O-linked glycosylation, hydrolysis, oxidation, phosphorylation, sulfation, reduction, ubiquitination, therapeutic, marker, therapeutic, marker*, and *abundance*.
- 8 points for each co-occurrence in a single sentence of an action term stem along with a gene and chemical, or gene and disease, or chemical and disease.
- 50 points for abstracts that allude to additional relevant data that exists only in the full text of the article. The details of the software developed by CTD to analyze the likelihood of additional data appearing only in the full text are too complex to summarize here, but abstracts containing the words “affymetrix,” or “agilent”, for example, or phrases such as “eight genes”, or “signaling pathways”, or “gene expression profiles”, or “12 probes”, fall into the category of abstracts appearing to allude to additional data that exists only in the full text of the article.
- 10 points for each occurrence of the target chemical in the title.
- 5 points for the occurrence of the target chemical in the PubMed MeSH annotation.
- 5 points for each occurrence of the target chemical in the first sentence of the abstract.
- 3 points for each occurrence of the target chemical in the second, last or second-to-the-last sentence of the abstract.

Lucene-Based Ranking Algorithm

Lucene accommodates document- and field search-based scoring boosts; both were used in conjunction with this study. The criteria and weighting are as follows:

- 2 point document boost for abstracts appearing in one of the priority journals described above
- 10 point document boost for each gene identified by the recognition tools and also resident in CTD as term or synonym
- 5 point document boost for each co-occurrence in a sentence of an action term stem along with a gene and chemical, or gene and disease, or chemical and disease
- 50 point document boost for abstracts that allude to additional data that exists only in the full text of the article
- 10 point field weight for search of the target chemical in the abstract title
- 2 point field weight for search of the target chemical in the abstract text
- 5 point field weight for search of the target chemical in the abstract's PubMed MeSH annotation
- 5 point field weight for search of the target chemical in the first sentence of the abstract
- 3 point field weight each for search of the target chemical in the second sentence, last sentence, and second-to-the-last sentence in the abstract.
